# Crystallography Open Database – an open-access collection of crystal structures

**DOI:** 10.1107/S0021889809016690

**Published:** 2009-05-30

**Authors:** Saulius Gražulis, Daniel Chateigner, Robert T. Downs, A. F. T. Yokochi, Miguel Quirós, Luca Lutterotti, Elena Manakova, Justas Butkus, Peter Moeck, Armel Le Bail

**Affiliations:** a Institute of Biotechnology, Graiciuno 8, LT-02241 Vilnius, Lithuania; b CRISMAT-ENSICAEN, Université de Caen Basse-Normandie, Bd. M. Juin, 14050 Caen, France; c Department of Geosciences, University of Arizona, Tucson, Arizona 85721-0077, USA; d School of Chemical, Biological and Environmental Engineering, 315 Gleeson Hall, Oregon State University, Corvallis, OR 97331-2702, USA; e Departamento de Química Inorgánica, Facultad de Ciencias, Universidad de Granada, 18071 Granada, Spain; f Department of Materials Engineering, University of Trento, via Mesiano, 77-38050 Trento, Italy; g Portland State University, Department of Physics, PO Box 751, Portland, OR 97207-0751, USA; h Université du Maine, Laboratoire des Oxydes et Fluorures, CNRS UMR 6010, Avenue O. Messiaen, 72085 Le Mans Cedex 9, France

**Keywords:** Crystallography Open Database, COD, structural databases

## Abstract

The Crystallography Open Database (COD), which is a project that aims to gather all available inorganic, metal–organic and small organic molecule structural data in one database, is described. The database adopts an open-access model. The COD currently contains ∼80 000 entries in crystallographic information file format, with nearly full coverage of the International Union of Crystallography publications, and is growing in size and quality.

## Abstract 2

The Crystallography Open Database (COD) is an ongoing initiative by crystallographers to gather all published inorganic, metal–organic and small organic molecule structures in one database, providing a straightforward search and retrieval interface. The COD adopts an open-access model for its >80 000 structure files.

## 1. Introduction

The Crystallography Open Database (COD) is a recent tool offered to the scientific community on the Web at http://www.crystallography.net/. It was founded in February 2003 as a response to Michael Berndt’s letter published in the Structure Determination by Powder Diffractometry (SDPD) mailing list (http://tech.groups.yahoo.com/group/sdpd/message/1016).

The historical fragmentation of structural data into three databases covering inorganic compounds (Inorganic Crystal Structure Database; Belsky *et al.*, 2002 ▶), metals, alloys and intermetallics (Crystal Data for Metals Database, CRYSTMET; White *et al.*, 2002 ▶), and organic and organometallic small molecules (Cambridge Structural Database; Allen, 2002 ▶) reflects the fact that in the past most public or private research laboratories concentrated their activity on one or the other of these very specialized topics. However, nowadays researchers at a given laboratory frequently extend their activities to all classes of compounds, focusing instead on materials with specific properties within more general classes of compounds, such as nanomaterials, hybrids or gas storage. Consequently, there is now a need to have access to all these databases simultaneously [together with powder data from the International Center for Diffraction Data (Kabekkodu *et al.*, 2002 ▶) that are generally added for identification purposes].

Thus, a group of scientists (Armel Le Bail, Luca Lutterotti and Lachlan Cranswick) responded quickly to Michael Berndt’s letter and teamed up to create an open crystallography database. The group contacted Professor Robert T. Downs who generously offered strong support for the concept, including the data set from the American Mineralogist Crystal Structure Database (AMCSD; Downs & Hall-Wallace, 2003 ▶; Downs, 2008 ▶), along with MySQL/PHP scripts written by Hareesh Rajan. At the same time, Daniel Chateigner joined, and less than three weeks after the letter from Michael Berndt, the COD project was announced through various Internet media (newsgroups, various mailing lists and what’s new pages).

This announcement introduced some new members to the effort (Brian Toby and Alexandre Yokochi) and, by the end of March 2003, the number of entries in the COD had increased to more than 5000. In order to ensure quality and standardization of uploaded files, the *CIF2COD* computer program was built by modifying *CIF2SX* with permission from Louis Farrugia. The first COD search page was coded in the PHP language. Uploads of crystallographic information files (CIFs) continued in April 2003 (1200 files from the Institut de Physique de la Matière Condensée, Grenoble) and after four months the number of entries in the COD surpassed 12 000 (Le Bail, 2003 ▶) as a result of uploads by individuals, laboratories and data shared by the AMCSD.

In December 2003, a subset of the COD was created and named PCOD (Predicted Crystallography Open Database) with the goal of gathering computationally predicted structures and with the expectation that the number of predicted entries could easily exceed the number of experimentally determined ones. In January 2004, the PCOD offered 200 entries.

By October 2005, the COD contained 20 000 entries. The database also had 30 new volunteers along with three new COD Advisory Board members (Saulius Gražulis, Miguel Quirós Olozábal and Peter Moeck). With the help of these volunteers, the number of entries in the COD increased to 48 000 by December 2006, now including 10 000 structures from the AMCSD (Downs & Hall-Wallace, 2003 ▶; Downs, 2008 ▶). In February 2007, a massive PCOD update boosted the number of entries to more than 60 000, with the help of the *GRINSP* software (Le Bail, 2005 ▶) for crystal structure prediction. At the same time, using the PCOD data, the Predicted Powder Diffraction Database (P2D2; Le Bail, 2008 ▶) was created, which provides identification by a search–match procedure similar to that of the Powder Diffraction File (Kabekkodu *et al.*, 2002 ▶).

In September 2007, the IUCr Executive Committee decided that the CIFs associated with structural papers published in IUCr journals should be made freely available to all databases, including the COD, giving the COD permission to routinely download new files from the IUCr site. This very welcome decision brought about a reorganization of the COD with the center of operations being transferred from Le Mans (France) to Vilnius (Lithuania) in December 2007.

Five years after its foundation, in 2008, the COD passed a major milestone by archiving the 50 000th entry, while PCOD climbed over the 100 000 structure limit in the same year. Our actions to date are but the start of this database, and the COD hopes that more crystallographers will upload their results in order to accelerate its completion.

New developments at the COD including automation of data deposition, data validation and correction, a novel search interface, and mirror sites and their synchronization, as well as the calculation of powder diffraction patterns, are briefly described in this paper.

## 2. Methodology

### 2.1. COD and PCOD contents

The COD and PCOD each consist of two major parts: an SQL database and a collection of structure data files. The structure files record crystallographic data that were published in peer-reviewed scientific journals, or that were determined or predicted and donated by established crystallographic laboratories. The master copy of the data is recorded in CIF format (Hall *et al.*, 1991 ▶).

From the master copy of the (P)COD data collection, data tables for the (P)COD SQL databases are generated. These tables abstract the most important crystallographic, chemical and bibliographic information and are used for online searches. Currently, the data tables contain cell constants (*a*, *b*, *c*, α, β, γ), cell volume, Hermann–Mauguin space-group symbol, a summary chemical formula, the number of distinct chemical elements, and a descriptive text that includes the chemical names of the substance and bibliographic references. A special field, coeditor code, is also included in the database, in order to generate URL links to the original papers for those journals that accept data sharing (currently, the IUCr journals).

We check and, when possible, restore systematic and trivial names of the reported chemical compounds and their formulas (IUPAC, structural and summary), since this information is vital for identification of the material. Information about chemical and hydrogen bonds is preserved in the COD CIFs if present in the original data file, but is not otherwise inferred from the structure.

Each structure deposited in the COD and the PCOD gets a unique seven-digit number, a (P)COD identifier. If a structure of a compound is redetermined, with higher precision or under different conditions, it will be deposited in the (P)COD under a new (P)COD number.

Since the COD identifier of a structure, once assigned, remains unchanged, a problem might arise when a deposited COD file needs to be changed for some reason, say, a syntax or data error must be corrected after the deposition. Currently, we have adopted a version control system called *Subversion* (Collins-Sussman *et al.*, 2008 ▶). Each change of any COD file is recorded in a central COD repository, and the new version of the file automatically gets a new revision number. These numbers, along with the COD repository address, are inserted by the software into the COD file header. There is a publicly accessible interface to the COD that allows older revisions of any file to be extracted and the COD change logs recorded by COD maintainers to be read. Having a COD number and the revision number of a file, it is always possible to restore a previous version of that file.

Structures are accepted in two formats – standard CIF format (http://www.iucr.org/resources/cif/) and a very simple REF (http://www.crystallography.net/ref.html) format, devised by A. Le Bail. The REF format is intended to be used in those cases where old data, predating the CIF era, need to be keyed in by hand or converted from some other format.

### 2.2. COD deposition procedure and validation

**Figure 1:**
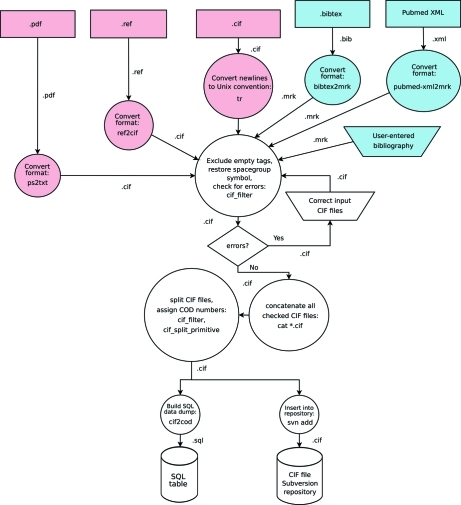
The COD deposition procedure. In this data flow diagram, circles indicate automatic processes and arrows show the data paths. As in control flow diagrams, a trapezoid indicates manual processes and a rhomb indicates a process where a decision to divert data *via* different paths is taken. Names after the colons in each node are the names of the Unix tools or COD-specific programs that were used for that operation. Rectangles are abstract (web) data sources – data sources depicted in pink provide crystallographic and chemical information (coordinates, symmetry data, formulae), while those depicted on a blue background provide bibliographic data. Cylinders denote internal COD disk storage facilities (databases). File extensions indicate file formats used. The .mrk file format is an intermediate format similar to XML designed for ease of parsing and editing, and used only internally by the COD deposition scripts.

Data in REF format, or occasionally in some other formats, are converted into the CIF format and then enter the same validation and deposition procedure as CIFs (Fig. 1 ▶). Each CIF is checked for syntax errors, using both the publicly available ‘vcif’ tool from the IUCr (McMahon, 1998 ▶) and our own CIF parser written in Perl (http://www.perl.com/; Wall *et al.*, 2000 ▶). Syntactic errors, if any, must be corrected manually; this task is currently performed by a COD maintainer responsible for deposition. When the syntax is correct, structures are assigned a new range of sequential COD numbers. Bibliographic information is taken either from the data sections of the CIFs, from the data_global sections, or from auxiliary files in BibTeX (Patashnik, 2003 ▶) or PubMed XML format (http://www.ncbi.nlm.nih.gov/entrez/query/static/overview.html). As a last resort, the bibliographic information may be taken from the names of the directories containing CIFs, which are then chosen to reflect journal, year and journal issue, or the name of the donating person and laboratory. Each separate CIF is given a full copy of available bibliographic information, so that it can be further processed and stored independently.

The CIFs now can be validated to check whether all necessary data items such as cell parameters, symmetry or bibliography are present. When all quality checks are passed, the existing COD database is scanned for duplicates. Duplicate structures are as a rule not deposited into the COD. A structure is considered a potential duplicate if its cell constants are within 0.5 Å and cell angles within 1.2° of any existing entry, the summary chemical formulas match, and both structures have been published in the same paper. If pressure and temperature are specified, these are also checked, and structures are considered duplicates only if they were measured under identical conditions. All potential duplicates are flagged and reviewed manually.

The final step involves insertion of the CIFs into the *Subversion* repository and insertion of the data dump into the COD SQL table. The checked CIFs are presented to the *CIF2COD* program, which computes some derived data and creates a data dump that can be loaded into the COD MySQL table. The new structures become available on the Web immediately after deposition.

Automated procedures have been developed to simplify the submission of data for users. For several years now, the *MAUD* software (Lutterotti *et al.*, 1999 ▶) has included algorithms for submission. Such functionality makes it simple to submit data to the COD; submission does not even require a visit to the COD Internet page. The ‘Submit Structure to COD’ submenu lists the CIF of one of the actual phases of a given analysis. The corresponding window allows manual modification of the file, if necessary, before the submission is completed by simply clicking the ‘Submit to COD’ button. After submission, the uploaded CIF is treated identically to other ‘regular’ submissions from the Internet.

## 3. Discussion

### 3.1. Current status of COD and PCOD

Currently, the COD stores over 80 000 structures of small organic and metal–organic molecules, inorganics, and minerals. The PCOD contains over 100 000 predicted inorganic crystal structures in CIF format, generated by the *GRINSP* programs (Le Bail, 2005). The number of different structure types is close to 30 000, the total number being attained by adding series of isostructural virtual compounds. For instance, there are ∼6400 different (Al/P)O_4_ compounds, and three other series of isostructural compounds with formulations SiO_2_, (Al/Si)O_4_ and (Al/S)O_4_. Besides the possibility of searching through the PCOD web interface, in a similar way to the COD, 48 complete series of compounds (characterized by the presence of the same chemical elements) are downloadable for prospective research.

For anybody who wishes to use the COD and the PCOD databases, the collected files are presented using standard open protocols and formats. The database can be searched online on the COD server using a simple web-based search form, and the structural results can be downloaded either one by one or in a compressed .zip file. Alternatively, the whole collection of the COD files and database tables can be downloaded from the COD web site (using the http protocol) as a compressed .zip, .tar.gz or .tar.bz2 file, or updated *via* an rsync protocol (http://samba.anu.edu.au/rsync/) from rsync://www.crystallography.net/cod-cif and rsync://www.crystallography.net/pcod-cif so that the files can be used and examined on a user’s local machine. Finally, the COD and PCOD CIFs, database dumps and web scripts are available for anonymous checkouts from the COD *Subversion* server (svn://www.crystallography.net/cod and svn://www.crystallography.net/pcod). From this server an interested user can reconstitute locally the whole COD database and the web site for local searches, and also browse COD deposition logs and retrieve older revisions, should they be necessary.

To facilitate the use of the COD as a reference database, it is planned that all data published in the COD will be assigned persistent URLs. Thus, any structure deposited in the COD should be available as http://www.crystallography.net/cif/〈COD number〉, *e.g.* http://www.crystallography.net/cif/1000000.cif.

The open-access nature of the COD and the PCOD permits the creation of numerous mirrors of the COD and the PCOD. At present, three mirrors are available at http://cod.ibt.lt/, http://cod.ensicaen.fr/ and http://nanocrystallography.org/. Currently, one centralized repository is kept as an authoritative source of data, but with the growth of the databases a decentralized implementation is possible.

### 3.2. Future directions of COD and PCOD development

A current challenge for all crystallographic databases, including the COD, is an exponential increase in the number of determined structural data entries. Fortunately, there is plenty of room to improve the efficiency of the COD deposition procedure. The current procedure involves a step in which a COD number is assigned by COD coordinator, and a step where the structures are checked by human depositors for possible errors. Both steps can be automated and parallelized. Finally, the structures still requiring human attention can be checked and edited in parallel by numerous COD reviewers all over the world, provided there is adequate software and enough volunteers participate in COD maintenance. Currently, the number of people contributing or willing to contribute to the development of the COD amounts to several dozen, apparently enough to provide qualified peer-review for the incoming structures. The development of the automatic data submission, annotation and CIF correction software is under way. Calculation of powder patterns is implemented for the PCOD data in the *Match!* software (http://www.crystalimpact.com/match/match18.htm).

For researchers who wish to publish their structure-related work, most journals require the deposition of structures with a crystallographic database and ask for the database accession number as proof of deposition. For such structures, a special deposition status, ‘on hold until publication’, will be introduced. The structures submitted to the COD with the ‘on hold’ flag will be included in the COD SQL database where their cell constants, composition, symmetry and authorship will be indicated. A COD number will be assigned to the structure and returned to the author, and will be visible through the search interface of the COD. The atomic coordinates themselves, however, will not be released to the public until either the publication describing them appears, the authors inform the COD team that the coordinates should be released, or one year elapses from the original deposition of the CIFs. If the structure is not published within one year, an e-mail will be sent to the depositing author asking whether the structure should be released or withdrawn.

At present, one of the main limitations of the functionality of the COD is the absence of a substructure search engine. In organic and metal–organic chemistry, the best way of defining such similarity is generally the presence of a common group of atoms chemically linked in the same way: this is what we call a ‘substructure’. For performing this task with COD data, we need to represent the chemical connectivity of the structures included in the COD in a suitable format, provide a tool for the user to input into the COD the definition of the substructure and finally employ a search–match engine that compares the user input against the COD data. A specialized chemical format such as CML (http://en.wikipedia.org/wiki/Chemical_Markup_Language) or SMILES (http://www.opensmiles.org/) with molecules already ‘grown’ across any possible crystallographic symmetry elements and simplifying the possible presence of chemically identical but crystallographically different moieties could be used for encoding the necessary information. The tools for user-friendly structure input and search are available under both free and commercial licenses (http://xdrawchem.sourceforge.net/, http://www.cambridgesoft.com/software/ChemOffice/, http://sourceforge.net/projects/joelib/, http://sourceforge.net/projects/cdk/). The remaining task is the integration of these tools with the COD.
